# New Insights into Glioblastoma

**DOI:** 10.3390/ijms25074090

**Published:** 2024-04-07

**Authors:** Eugenia Cella, Alberto Bosio, Giuseppe Lombardi

**Affiliations:** 1 Department of Oncology, Oncology 1, Veneto Institute of Oncology IOV-IRCCS, 35128 Padua, Italy; eugenia.cella@hotmail.it (E.C.); giuseppe.lombardi@iov.veneto.it (G.L.); 2 Department of Internal Medicine and Medical Specialties (DiMI), School of Medicine, University of Genova, 16132 Genova, Italy

Glioblastoma (GBM) is the most aggressive malignant primary central nervous system (CNS) tumor and, despite decades of research, it remains a lethal disease with a median overall survival of less than two years [1]. Different treatment strategies have been investigated for newly diagnosed and recurrent GBM, but unfortunately, about 90% of patients relapse and no treatment has shown clinically meaningful results [2]. Hence, the investigation of novel therapies and the comprehension of GBM heterogeneity and biological processes are a clinical priority.

The aim of this Special Issue is to highlight the clinical impact of novel therapies in the treatment of GBM, with a particular focus on their molecular aspects.

Sphingolipids play a role in oncogenesis by modulating the cell membrane composition. Navone et al. [3] demonstrated the clinical utility of luteolin, a natural flavonoid, as an anti-tumor regulating sphingolipids’ signaling transduction. In a complementary phase 1/2a trial, Lopez et al., 2023 [4] showed the acceptable tolerability and promising clinical activity of the synthetic hydroxylated lipid acid (2-OHOA), which has been designated by the European Medicines Agency (EMA) and the Food and Drug Administration (FDA) as an orphan drug for the treatment of GBM.

Another factor involved in GBM treatment failure is the blood–brain barrier (BBB), which is an obstacle in delivering optimal concentrations of anti-tumor drugs [5]. The use of devices emitting low-intensity pulsed ultrasound waves with the concomitant administration of intravenous microbubbles (LIPU-MB) is under investigation in order to evaluate its emerging role in opening the BBB. Sonabend et al. [6] conducted a dose-escalation phase I trial with nab-paclitaxel in 17 patients with recurrent GBM, investigating the efficacy of a skull-implanted ultrasound device in transiently opening the BBB and, thus, delivering a higher drug concentration into the tumor. It emerged that LIPU-MB can effectively enhance drug delivery across the BBB while maintaining an acceptable toxicity profile, with transitory headaches being the most frequent adverse event experienced. The NCT 05902169 is an ongoing randomized phase III trial designed to confirm the efficacy of LIPU-MB in opening the BBB with concurrent carboplatin versus standard chemotherapy for the first recurrence of GBM.

Another challenging topic in treating GBM is represented by its immunosuppressive nature. Unlike several other solid tumors, no promising results have been achieved with current immunotherapy strategies [7,8,9]. Indeed, GBM has a low mutational burden, and it is characterized by the secretion of paracrine molecules, with immunosuppressive activities. To overcome these factors, a deeper comprehension of GBM’s immune microenvironment and its crosstalk with inflammation mediators is needed. Tumor necrosis factor (TNF) is an immunocytokine mostly produced by activated macrophages, T lymphocytes and natural killer (NK) cells which has a strong antitumor effect and a powerful immunostimulatory effect [10]. Due to its intrinsic unacceptable toxicity at therapeutically effective doses, a recombinant form is being evaluated in a Phase II trial (NCT04573192) in combination with lomustine versus lomustine alone in first relapse GBM patients.

In oncology, there is currently a particular focus on the molecular characterization of tumors. Next-generation sequencing (NGS) is used to identify molecular alterations that are possibly susceptible to specific targeted therapies [11]. The recent 2021 World Health Organization (WHO) classification of CNS tumors refined the diagnosis of CNS tumors by integrating historical histological parameters with molecular profiles [12]. Among them, the molecular alterations with the greatest clinical impact in neuro-oncology, according to the ESMO Scale for the clinical actionability of molecular targets (ESCAT), are represented by the BRAF V600E mutation and NTRK fusions [13]. Wen et al. [14], in a phase 2 study (the ROAR trial), demonstrated the clinical activity and safety of dabrafenib plus trametinib in patients with BRAF V600E-mutated recurrent high- and low-grade gliomas.

Regarding the NTRK fusions, the results look promising as assessed by Doz et al. [15], with a meaningful clinical result using larotrectinib, especially in the setting of a pediatric population. After their publication, the 2023 European Association of Neuro-Oncology (EANO) guidelines emphasized the importance of including BRAF V600E and NTRK analysis in the diagnostic process, as they should be considered possible therapeutic targets for glioma patients [16].

Despite the large number of molecular alterations identified as potential targets, such as EGFR and MET amplifications and ROS1 and ALK fusions, few of them showed clinical relevance when administered as monotherapy [17]. However, most of the trials analyzing these specific targeted therapies, such as FGFR and MET alterations, are ongoing, and results are expected in the next few months. In addition, it will be extremely important to collect data in a prospective international registry.

A possible therapeutic strategy is to use nonselective inhibitors, such as regorafenib, a multikinase inhibitor with antiangiogenic activity. Regorafenib showed promising results in REGOMA, a randomized trial comparing regorafenib to lomustine in patients with recurrent GBM; subsequently, GBM AGILE showed no benefit for regorafenib over lomustine in this setting [18,19,20]. Therefore, the use of biomarkers associated with regorafenib efficacy will be necessary to identify responders. A dose-escalation Phase I trial (NCT06095375) of regorafenib in combination with radiochemotherapy with temozolomide is ongoing in patients with newly diagnosed GBM.

The management of GBM still remains an ongoing challenge, despite significant research efforts. The diverse therapeutic strategies discussed in this Special Issue highlight the complexity of GBM treatment and the need for multifaceted approaches to improve patient outcomes.

Interdisciplinary efforts encompassing molecular biology, drug delivery technologies, and immunotherapy represent promising avenues for advancing GBM treatment. Indeed, continued research, including ongoing clinical trials, is essential to translate these findings into meaningful clinical outcomes and ultimately improve patient outcomes.
